# Tear Film Imager Findings Support a New Perspective on the Mechanisms of Tear Film Thinning in Healthy Subjects

**DOI:** 10.1167/iovs.67.5.22

**Published:** 2026-05-11

**Authors:** Michel M. Sun, Camille Beniga, Suzanne Zheng, Scott Gladstein, Francisco J. López, Xiaoming Xu, Ashley Nguyen, Michael R. Robinson

**Affiliations:** 1 AbbVie, Irvine, California, United States; 2 AbbVie, North Chicago, Illinois, United States

**Keywords:** healthy volunteers, interferometry, meibomian gland dysfunction (MGD), tear film dynamics, tear film interferometry, tear film thinning, tear meniscus, tears

## Abstract

**Purpose:**

Tear film thinning primarily occurs by two mechanisms: evaporation and tangential redistribution. The Tear Film Imager (TFI; AdOM, Israel) was used in healthy subjects to simultaneously measure multiple tear film parameters through normal blink cycles to bring a new perspective on the relative contributions of these mechanisms to tear film thinning.

**Methods:**

Tear film lipid layer thickness (LLT), muco-aqueous layer thickness (MALT), and inter-blink muco-aqueous layer thinning rate (MALTR) were measured with the TFI in 20 healthy subjects. Tear film evaporation rates were obtained from a PubMed literature review.

**Results:**

Tear film geometric mean (geometric standard deviation) measurements in healthy subjects were 52.7 (1.55) nm for LLT (*n* = 20), 3282 (1.3) nm for MALT (*n* = 20), and −63 (2.0) nm/s for MALTR (*n* = 17). Literature-based mean tear film evaporation rates in healthy subjects, measured using either closed chamber evaporimeters or ventilated chambers allowing air flow across the ocular surface, ranged from 4.07 to 39.3 nm/s (weighted mean = 11.3 nm/s).

**Conclusions:**

In healthy subjects, the inter-blink MALTR measured by the TFI is too rapid to be accounted for by reported rates of tear film evaporation. The MALTR in the early period after blinking appears to result mainly from tangential flow and redistribution of the muco-aqueous layer, predominantly driven by the Marangoni effect, with gravity and other forces playing a comparatively minor role. Increased understanding of the mechanisms of tear film thinning and break-up time in healthy subjects may potentially help guide the development of novel therapeutic options for dry eye.

The tear film is a complex and dynamic thin fluid layer covering the ocular surface.^1^ Its functions include protecting and lubricating the ocular surface to preserve the health and function of the cornea and conjunctiva, and providing a smooth optical surface for light refraction.^2^ An intact tear film is required for clear and comfortable vision.^3^

The tear film has a laminar structure consisting of an inner muco-aqueous layer on the ocular surface, and a thinner tear film lipid layer (TFLL) lying superficially at the tear-air interface.^3^ The layers of the tear film move and are continuously being renewed. With each blink, movement of the upper lid spreads the tear film across the ocular surface.^4^ The spreading of the TFLL is driven by the surface tension gradient, which is related to the structure and viscoelastic properties of the TFLL.^5^ The rheology and viscoelastic properties of the TFLL influence both the TFLL spread rate and the capability of the TFLL to provide mechanical stability of the air/tear surface and resistance to tear film break-up during the interblink period.^6^ TFLL functions include reducing the surface tension of the tear film at the tear-air interface, helping the tear film spread over the ocular surface, serving as a barrier to evaporation, and maintaining tear film stability.^5^^–^^7^ The tear film spreading and evaporative barrier functions of the TFLL are not mutually exclusive; both occur simultaneously and are facilitated by a thick and uniform TFLL.

The central tear film thins progressively during the inter-blink interval but is replenished with the next blink.^4^ This thinning of the precorneal tear film has generally been believed to result mainly from evaporation with other mechanisms, including tangential flow along the corneal epithelium and gravitational pull of the aqueous layer downward to the inferior meniscus, also involved.^8^^–^^11^ Precorneal tear film thinning in the inter-blink interval is a normal physiological process that occurs in healthy eyes and leads to tear film instability and break-up.^12^ Acceleration of the rate of tear film thinning results in rapid tear film break-up time, which is a hallmark feature of dry eye disease (DED).

As defined by the Tear Film and Ocular Surface Society Dry Eye WorkShop II (TFOS DEWS II), DED is a multifactorial disease of the ocular surface characterized by loss of homeostasis of the tear film and accompanied by ocular symptoms, with tear film instability (usually measured by tear film break-up time), hyperosmolarity of the tear film, ocular surface inflammation and damage, and neurosensory abnormalities playing etiological roles in the disease.^13^ DED can be classified as aqueous-deficient, evaporative, or mixed.^13^ Production of the aqueous component of tears is markedly reduced in aqueous dry eye, whereas evaporation from the ocular surface is increased in evaporative dry eye. In evaporative DED, conventional belief is that increased evaporation from the tear film results in a more rapid rate of tear film thinning between blinks, leading to tear film instability and break-up. Evaporative DED is commonly associated with meibomian gland dysfunction (MGD).^14^ The TFLL is largely composed of meibum, a complex mixture of lipids secreted from the meibomian glands located in the upper and lower eyelids. In MGD, decreases in the quality (composition) and/or quantity of the secreted meibum result in changes in the structure of the TFLL and decreased TFLL function.^7^

The Tear Film Imager (TFI; AdOM, Israel) is a non-contact clinical diagnostic device that uses spectral interferometry to measure the individual layers of the tear film with nanometer resolution and allows assessment of changes in the tear film over time.^15^^–^^18^ Uniquely, this device is able to simultaneously measure the thickness and dynamic changes of both the muco-aqueous layer and the lipid layer of the tear film. During the examination, a 40-second scan of the tear film overlying the central 6 mm of the cornea is taken while the subject blinks normally. Muco-aqueous layer thickness (MALT) and lipid layer thickness (LLT) measurements are taken as frequently as 10 times each second of the scan.^15^ The TFI may be a valuable tool in understanding the complex behavior of the tear film in health and disease because of its ability to capture dynamic changes in the individual tear film layers while tear film dynamics are maintained by normal blinking.^16^

A comprehensive understanding of the mechanisms involved in tear film thinning, including the relative importance of evaporation and tangential redistribution, is lacking. This study obtained TFI data from subjects with healthy eyes. The findings have the potential to redefine current understanding of tear film dynamics and may be relevant to the etiology and treatment of DED.

## Methods

This study was conducted in accordance with the principles of the Declaration of Helsinki, and the study protocol was approved by an institutional review board at the study site before study initiation. All subjects provided written informed consent.

Tear film dynamics were evaluated with the TFI in healthy subjects at the AbbVie Eye Care Research Center (Irvine, CA, USA). Subjects were required to be ≥18 years of age and in good general health, with no history of diagnosis of DED or other medical or ophthalmic conditions that could affect interferometry measurements. Eligibility criteria included no ocular surface findings of greater than mild (1+) severity on slit-lamp biomicroscopy; no history of ocular surgery; no active or documented history of any condition requiring local or systemic therapy that could impact study assessments; and no history within the past 30 days of hospitalization, any surgical procedure, or clinically significant illness or infection. Subjects were not permitted to use artificial tears or contact lenses within 2 or 7 days, respectively, prior to the interferometry measurements.

**Figure 1.**
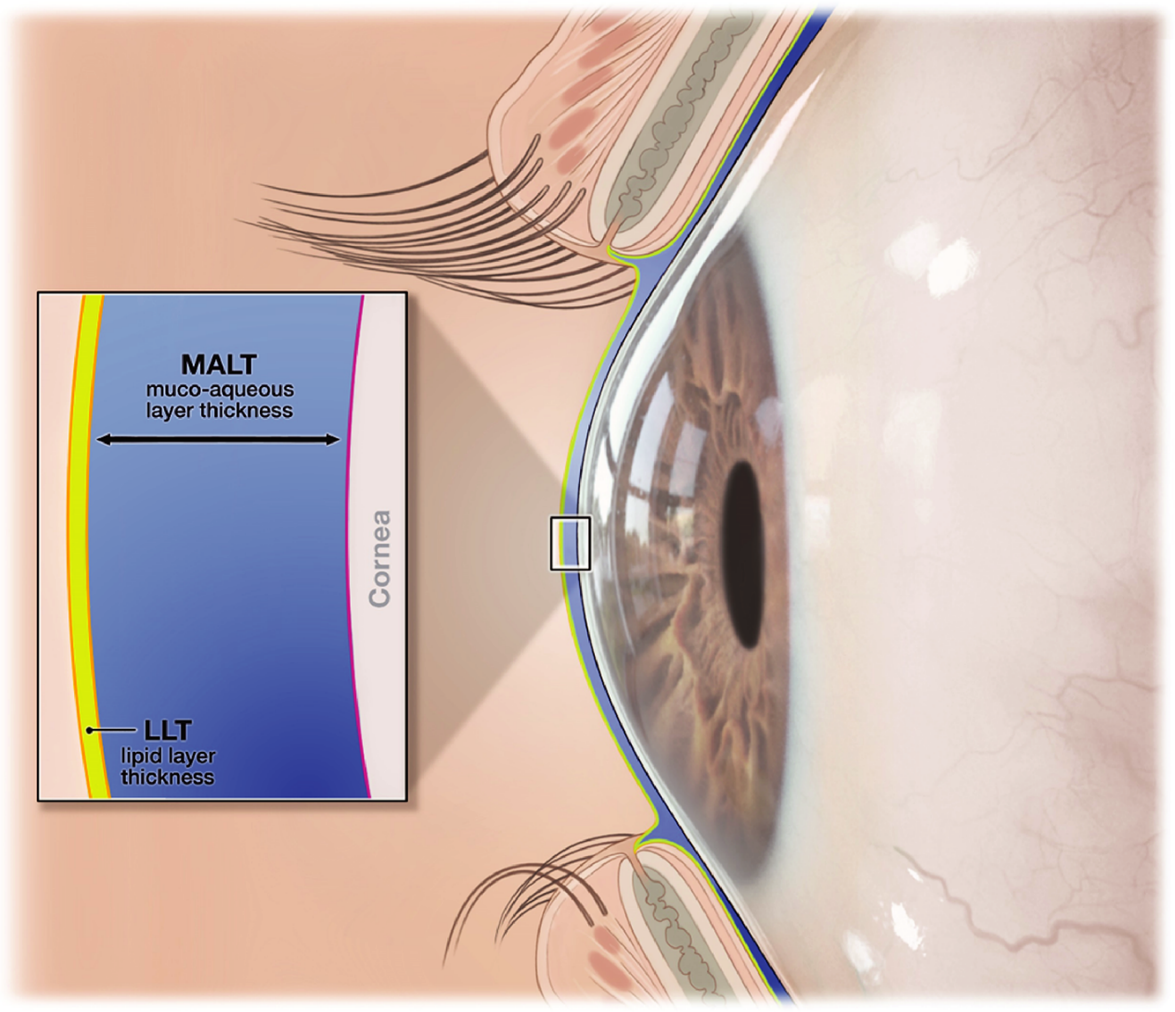
Diagram showing the muco-aqueous and lipid layers of the precorneal tear film. The parameters measured by the TFI include the muco-aqueous layer thickness (MALT) and the lipid layer thickness (LLT). TFI, Tear Film Imager.

Tear film interferometry was performed in the right (study) eye of subjects with the TFI. The TFI provides noninvasive, dynamic imaging of the tear film through normal blink cycles and uses proprietary software to produce measurements of LLT and MALT (Fig. 1) as well as muco-aqueous thinning rate (MALTR), inter-blink interval (IBI), lipid map uniformity (LMU), and lipid break-up time (LBUT).^16^ TFI measurements were taken at study visits on days 1 and 8 at baseline (before any intervention during the visit). At each visit, 2 separate sets of baseline measurements were taken no sooner than 10 minutes after the start of the visit (to allow stabilization of the tear film) and 45 minutes later.

All TFI measurements were taken on the same TFI instrument and in the same examination room. Temperature and relative humidity were monitored and recorded; at the time of the TFI measurements, the mean standard deviation (SD) relative humidity was 55.2% (5.50%) and the mean (SD) temperature was 71.8 (0.85)°F. The manufacturer's instructions for using the instrument were followed, and subjects were asked to blink normally during the measurements. The analysis used observed values averaged for each subject across all four sets of baseline measurements, with descriptive statistics calculated across the study population. These statistics included the geometric mean and geometric SD because the data were heavily skewed and approximated a log-normal distribution.

The rate of change in LLT (LLTR) was calculated using the same methodology used for calculation of MALTR. Each interblink interval was divided into seven segments, and the rate of LLT change within the interblink interval was determined by linear regression of LLT measurements in the middle five segments (excluding measurements just before and after a blink). LLTR was calculated as the average rate of LLT change across all interblink intervals in the TFI measurement.

### Literature Review

A literature review was conducted to determine reported tear evaporation rates. The search strategy (“tear” OR “ocular surface”) AND (“evaporation”) AND (“rate” or “rates”) with no time or language restrictions was used on June 1, 2025, to identify relevant articles in the PubMed database. Primary and review articles including data on human tear evaporation rates were selected for review. Relevant articles cited by the selected articles were also reviewed. Tear evaporation rates, the measurement techniques used, and characteristics of the study populations were extracted.

All evaporation values reported in units of g/m^2^/h were expressed in units of 10^−7^ g/cm^2^/s by multiplying by 0.2778 (or dividing by 3.6). The conversion from mass loss (evaporation) to thickness change then required only the assumption that the specific density of the evaporating fluid was 1 g/cm^3^ (the specific density of water at 4°C).

Linking the mass loss to volume change and assuming that the volume of 1 g is 1 cm^3^: 10^−7^ g/cm^2^/s = 10^−7^ cm^3^/cm^2^/s = 10^−7^ cm/s = nm/s. Therefore, tear film evaporation in units of 10^−7^ g/cm^2^/s represents tear film thinning in units of nm/s.

## Results

Twenty-one healthy adults with no history of DED were recruited for the tear film interferometry study. The subjects were White (11 [52.4%]) or Asian (10 [47.6%]), and two-thirds were women (14 [66.7%] women and 7 [33.3%] men). The mean (SD) subject age was 42.4 years (10.2 years). One subject was discontinued from the study due to inability to obtain adequate TFI scans.

**Figure 2.**
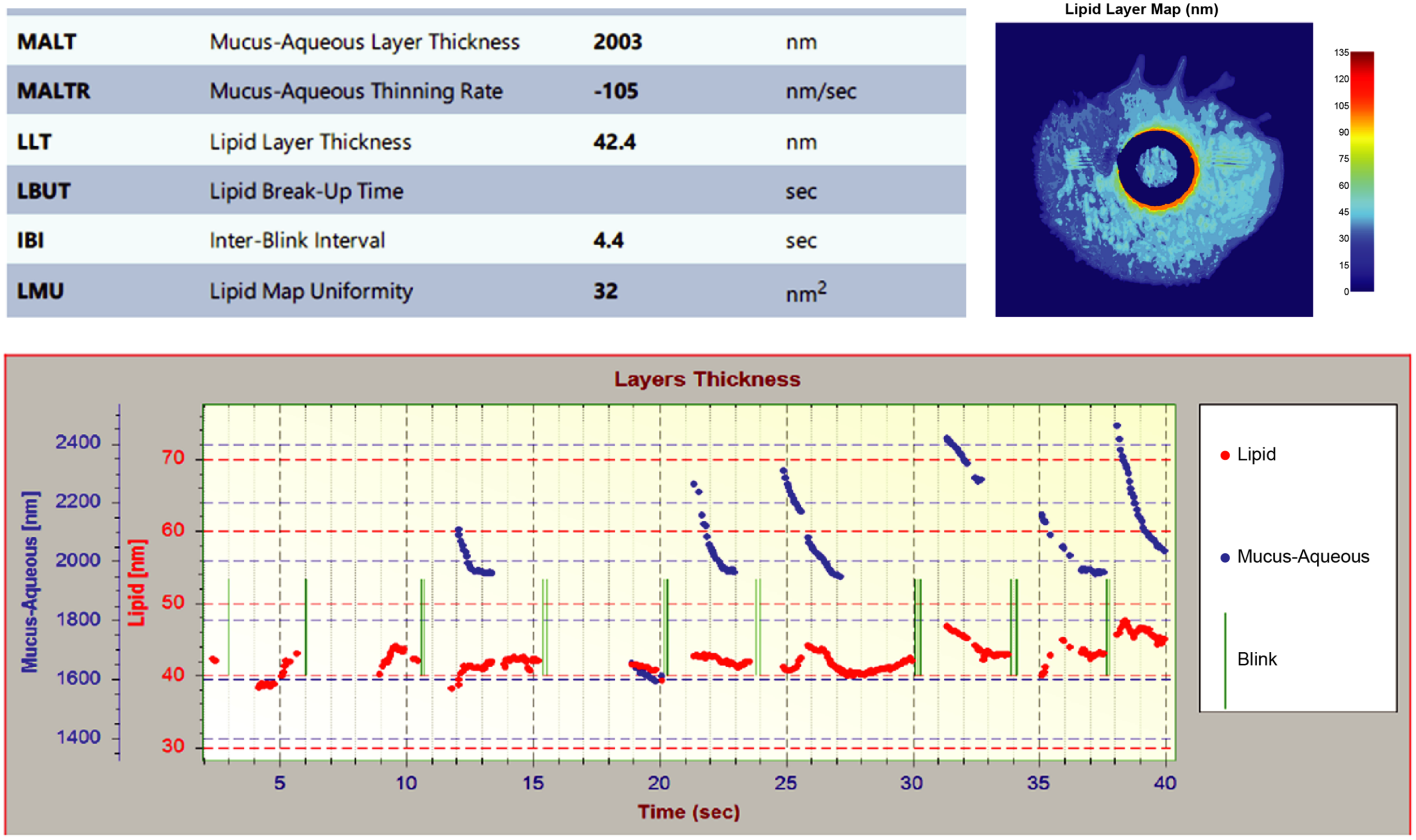
TFI interferometry data for a healthy eye. In the output (*top left*), MALT is the average of MALT measurements (shown in *blue* in the time-lapse graph) with measurements just before and after a blink excluded. MALTR is the average slope of the MALT time trend, also excluding MALT measurements just before and after a blink. LLT in the output (*top left*) is the average of LLT measurements (shown in *red* in the time-lapse graph). IBI, inter-blink interval; LBUT, lipid break-up time; LLT, lipid layer thickness; LMU, lipid map uniformity; MALT, muco-aqueous layer thickness; MALTR, muco-aqueous thinning rate; TFI, Tear Film Imager.

TFI scans were obtained for 20 healthy subjects. Figure 2 shows the TFI output for a representative subject. The time-lapse graph of tear film MALT and LLT measurements during normal blink cycles demonstrated dramatic thinning of the muco-aqueous layer during the inter-blink interval, whereas the LLT remained relatively constant. The mean (SD) LLTR was −0.03 (2.6) nm/s (95% confidence interval [CI] = −1.25 to 1.18; median = −0.24, *n* = 20).

The results of the TFI assessments are summarized in Table 1. The TFI output included LLT and MALT for all 20 subjects with usable scans and MALTR for 17 subjects. Geometric mean (geometric SD) values were LLT = 52.7 (1.55) nm (*n* = 20); MALT = 3282 (1.3) nm (*n* = 20); MALTR = −63 (2.0) nm/s (*n* = 17); IBI = 6.1 (1.77) seconds (*n* = 20); LBUT = 3.8 (2.00) seconds (*n* = 14); and LMU = 42 (3.5) nm^2^ *(*n = 20; see Table 1).

**Table 1.** Tear Film Interferometry Measurements in the Study Eye of Healthy Subjects

Parameter	*n*	Mean (SD)	Min, Max	Median (25%, 75% Percentiles)	Geometric Mean (Geometric SD)	95% CI of Geometric Mean	Range Calculated Using Geometric SD
LLT, nm	20	58.2 (28.81)	27.7, 133.0	47.8 (39.4, 71.6)	52.7 (1.55)	42.86 to 64.75	33.9 to 81.9
MALT, nm	20	3404 (963.6)	2210, 5075	2942 (2644, 4123)	3282 (1.3)	2885.7 to 3732.0	2486 to 4332
MALTR, nm/s	17	−80 (59.3)	−229, −24	−52 (−101, −34)	−63 (2.0)	−87.2 to −45.3	−127 to −31
IBI, s	20	7.3 (5.18)	2.7, 24.0	5.3 (3.9, 8.5)	6.1 (1.77)	4.65 to 7.94	3.4 to 10.8
LBUT, s	14	4.9 (4.22)	1.3, 17.7	3.1 (2.8, 5.9)	3.8 (2.00)	2.77 to 5.31	1.9 to 7.7
LMU, nm^2^	20	120 (264.4)	11, 1171	31 (17, 58)	42 (3.5)	23.5 to 75.6	12 to 147

CI, confidence interval; IBI, inter-blink interval; LBUT, lipid break-up time; LLT, lipid layer thickness; LMU, lipid map uniformity; MALT, muco-aqueous layer thickness; min, minimum; MALTR, muco-aqueous thinning rate; max, maximum; SD, standard deviation.

The intraclass correlation coefficient for the 2 sets of baseline TFI measurements taken at each visit generally were in the range of 0.5 to 0.89 (Table 2), indicating fair to good within-subject repeatability.^19^ However, the MALTR measurements showed poor within-subject repeatability between visits (see Table 2).

**Table 2.** Repeatability of the Tear Film Imager Measurements

	Short-Term Repeatability During the Study Visit, From Set 1 to Set 2		Longer-Term Repeatability From Day 1 to Day 8	
Parameter	Number of Paired Measurements	ICC (95% CI)	Number of Paired Measurements	ICC (95% CI)
LLT	40	0.8185 (0.6463 to 0.9046)	40	0.7427 (0.5095 to 0.8619)
MALT	38	0.6507 (0.4287 to 0.8113)	37	0.6265 (0.3516 to 0.7759)
MALTR	16	0.5366 (0.1196 to 0.8067)	15	0.2204 (0.0000 to 0.5985)
IBI	40	0.8486 (0.7203 to 0.9222)	40	0.6852 (0.4175 to 0.8320)
LBUT	6	0.918^*^	6	0.412^*^
LMU	36	0.8562 (0.7028 to 0.9286)	35	0.8131 (0.6070 to 0.9012)

ICC, intraclass correlation coefficient.

Repeatability was assessed using log-transformed data.

* The 95% CI was not calculated because the bootstrap method used for the calculation requires a larger number of observations for reliable estimates.

### Tear Evaporation Rates in the Literature

Studies that reported tear film evaporation rates measured in units of 10^−7^g/cm^2^/s or g/m^2^/h in healthy eyes were reviewed, and the evaporation rates were converted to units of nm/s thinning rates of an aqueous film (Table 3). The weighted mean evaporation rate across the studies (involving a total of 345 healthy subjects) was 11.3 nm/s, with mean evaporation rates measured in the individual studies ranging from 4.07 to 39.3 nm/s (see Table 3). The lowest evaporation rate (4.07 nm/s) was reported in a study by Rolando and Refojo^20^ who measured evaporation from the ocular surface by the change in relative humidity of the air in a closed chamber system (fitted goggles over the test eye containing air with 29.5% humidity at baseline) over a 1-minute period. During testing, the subject was asked not to blink, and the eye lids and skin under the goggles were covered with petroleum jelly to prevent evaporation from nonocular surfaces. The highest evaporation rate (39.3 nm/s) was reported by Liu et al.,^21^ who used a ventilated chamber system consisting of an eyecup covering the eye with air (of known humidity) infused into the cup at a constant flow rate of 150 mL/min and outflow to a humidity sensor. Evaporation was measured by the difference between the water content of the air entering and exiting the chamber, and the ocular surface evaporation rate was derived by subtracting the evaporation rate measured with the eye closed from the evaporation rate measured with the eye open and adjusting for the areas of the ocular surface and skin (lids) within the eyecup. The literature search revealed one study that reported an ocular surface evaporation rate higher than 39.3 nm/s in subjects who were not identified as having DED.^22^ This study used a ventilated system in which a probe with air infused at a constant flow rate of 150 mL/min was attached to a goggle, and an experimental humidity sensor with an epoxy-coated quartz crystal sensor surface was used to measure evaporation by measuring the difference between the water content in the air entering and exiting the probe. The reported rate of evaporation was 83.06 nm/s, but the characteristics of the study subjects were not reported, and it is possible that they had ocular surface disease.^22^ Also notably, the relative humidity of the input air to the probe was only 10%, which might have increased the evaporation from the ocular surface.

**Table 3.** Tear Evaporation Rates Reported in Healthy Subjects

Study	*N*	Evaporation Rate, nm/s^*^ Mean (SD)
Rolando and Refojo, 1983^20^	52	4.07 (0.40)
Tomlinson and Giesbrecht, 1994^38^	21^†^	8.19 (5.9)
Tomlinson and Giesbrecht, 1994^38^	26^‡^	12.2 (6.8)
Mathers, 1993^39^	20	14.8 (6)
Mathers et al., 1996^40^	72	15.1 (8.62)
Mathers and Daley, 1996^41^	34	13 (6)
Tomlinson et al., 2001^42^	9^§^	7.2 (3.3)
Goto et al., 2003^43^	22	4.1 (1.4)
McCulley et al., 2003^44^	22	7.29 (2.19)
Liu et al., 2005^21^	20^||^	39.3 (13.6)
Khanal et al., 2009^45^	32	5.83 (3.29)
McCann et al., 2009^46^	15	5.0 (3.0)
Total, weighted mean (pooled variance)^#^	345	11.3 (6.30)

* Evaporation rates reported in units of × 10^−7^g/cm^2^/s or g/m^2^/h are expressed as nm/s thinning rates of an aqueous film.

† Male subjects.

‡ Female subjects.

§ Female subjects on day 2 of their menstrual cycles.

|| Twenty eyes in 10 healthy subjects in the control arm of the study.

# For calculation of the weighted mean, the mean and number in each study were multiplied, and the products were summed and divided by the total number across all studies.

## Discussion

Tear film thinning is important in both healthy and disease states because it leads to tear film instability and break-up.^12^ Thinning of the tear film during the inter-blink interval has generally been believed to result mainly from evaporation.^8^^–^^10^ However, other mechanisms including tangential redistribution of the muco-aqueous layer from gravitational force downward and the Marangoni effect (the phenomenon in which variations in surface tension along a liquid-liquid or liquid-gas interface cause fluid movement from areas of lower surface tension to areas of higher surface tension) may also be important.^12^ In this study, the recently developed TFI interferometer was used to analyze tear film dynamics over the central cornea in a cohort of healthy subjects. The TFI time-lapse measurements showed that the tear film LLT remained relatively constant during the inter-blink interval, whereas the MALT decreased rapidly, suggesting that tear film thinning in healthy eyes is caused by isolated thinning of the muco-aqueous layer. An average (geometric mean) rate of muco-aqueous layer thinning of −63 nm/s was demonstrated in the study subjects. Comparison of this thinning rate with reported rates of tear film evaporation in healthy subjects suggests that evaporation may account for no more than approximately 18% to 62% of the muco-aqueous layer thinning. These results suggest that a mechanism other than evaporation (such as tangential redistribution) must be involved and may potentially be the primary driver of tear film thinning measured with the TFI.

Discrepancies between measured rates of tear film thinning or break-up time and evaporation rates have been long recognized. Nichols et al.^23^ used interferometry to measure tear film thinning in healthy eyes after a blink and reported a mean thinning rate of 63.2 nm/s. The authors compared these results with literature values for tear film evaporation ranging from 4.0 to 24.2 nm/s, and concluded that (1) evaporation could not account for the entirety of tear film thinning, and (2) tear film flow parallel to the ocular surface (tangential flow), which could be caused by gradients of surface tension in the tear film (Marangoni flow) or by curvature of the tear film surface that generates pressure gradients in the tear film, is also involved.^23^ In a subsequent article by the same group,^8^ the observed 63.2 nm/s thinning rate was compared with an average tear film evaporation rate of 12.5 nm/s from studies of ocular surface evaporation reviewed by Mathers.^24^ These values suggest that evaporation accounts for only approximately 20% of the thinning rate; however, the authors discussed the caveat that the average reported evaporation rate in the reviewed studies likely underestimated the evaporation that occurs under real-world conditions.^8^ Most of the studies evaluating evaporation rates used closed preocular chambers that prevent normal air flow over the corneal surface, which could allow a layer of humid air to build up over the ocular surface and impede evaporation.^8^ In a study reported by Liu et al.^21^ that used a ventilated chamber for the assessment, the mean evaporation rate in healthy eyes was 39.3 nm/s,^21^ a value that would account for approximately 62% of the tear film thinning rate observed in the study by Nichols et al.^23^ and approximately 62% of the mean geometric MALTR (76% of the median MALTR) observed in our study. The authors commented that under real-world conditions, evaporation may have a major role in tear film thinning in healthy eyes.^8^ Consistent with the concept that evaporation is a major contributor to tear film thinning, in a study by Kimball et al.^9^ that evaluated the tear film with interferometry, significant tear film thinning between blinks was measured in free air conditions but not when subjects wore air-tight goggles.

In our study, the geometric mean rate of tear film muco-aqueous layer thinning measured with the TFI in healthy eyes was 63 nm/s (geometric mean MALTR = −63 and median MALTR = −52). It is difficult to compare this value with the literature because of the scarcity of TFI studies evaluating MALTR in healthy eyes. However, Mangwani-Mordani et al.^17^ reported a median MALTR of −36 nm/s in healthy subjects (*n* = 16). In our review of tear film evaporation rates measured with a number of different methods involving closed and ventilated chambers, the mean evaporation rate in healthy eyes was 11.3 nm/s, and the highest rate of evaporation reported in healthy eyes was 39.3 nm/s.^21^ Comparing these values with the measured mean geometric MALTR in our study (−63 nm/s), we estimate that evaporation could account for only 18% to 62% of the muco-aqueous layer thinning that was observed, and another mechanism must explain the remaining 38% to 82% of the muco-aqueous thinning rate over the central cornea.

The tear menisci at the junctions of the bulbar conjunctiva and the margins of the upper and lower eyelids contain most of the tear fluid (estimated 75%–90%).^25^ Thus, the tear meniscus height (TMH) is commonly used as a measure of tear volume, with a measurement of 0.20 mm or lower diagnostic of aqueous deficiency.^26^ However, TMH may also be a useful surrogate measure of tangential tear film fluid flow to the menisci. In a study using video recordings in subjects without DED, the TMH of both the upper and lower menisci was demonstrated to increase significantly while the eye remained open during the 10 seconds after a blink.^27^ Another study using anterior segment optical coherence tomography (AS-OCT) to evaluate the effects of blinking on tear dynamics in healthy subjects showed that in subjects with delayed blinking, the decrease in tear film thickness during the inter-blink interval was accompanied by a significant increase in the TMH of both the upper and lower menisci.^11^ Results in subjects who were permitted to blink normally were qualitatively similar, but the increase in the TMH in the inter-blink interval was significant only for the TMH of the upper meniscus.^11^

TMH can be measured with the Keratograph 5M (OCULUS, Inc., Arlington, WA, USA). Studies have demonstrated that Keratograph 5M measurements of TMH are reproducible and similar to AS-OCT measurements of TMH in healthy eyes.^28^^,^^29^ We previously reported an observational study using the Keratograph 5M to measure TMH in subjects with and without MGD (Sun MM, et al. IOVS 2025;66:ARVO E-Abstract 5692). Subjects were assigned to non-MGD, mild/moderate MGD, and severe MGD cohorts (25/cohort) based on meibum quality evaluation, ocular symptoms, and Schirmer test results, and TMH was measured at a central location along the lower lid margin. The mean (± SD) TMH was 0.269 ± 0.088 mm in the non-MGD cohort, 0.346 ± 0.157 mm in the mild/moderate MGD cohort, and 0.434 ± 0.247 mm in the severe cohort MGD, with the mean TMH significantly higher in the severe MGD cohort compared with the non-MGD cohort (*P* = 0.034). The TMH measured in the non-MGD cohort was nearly identical to a previously reported mean TMH of 0.27 ± 0.12 mm in healthy eyes.^26^ Our finding that the central location TMH was significantly higher in the severe MGD cohort was consistent with results of two previous studies.^30^^,^^31^ In a study by García-Marqués et al.^30^ evaluating the ability of the Keratograph 5M to help diagnose MGD, the TMH measured in subjects with MGD (defined as meeting at least 2 of the following criteria): Ocular Surface Disease Index score ≥13; lid margin abnormality score >1; and meiboscore >1) was significantly higher than the TMH in subjects without MGD. In another study, Robin et al.^31^ evaluated meibography findings in patients with symptomatic ocular surface diseases and reported that the TMH of the lower meniscus was significantly higher in the cohort with MGD than in the cohort with other ocular surface diseases (perennial allergic conjunctivitis and primary and secondary Sjogren syndromes).

Potential explanations for increased TMH in patients with severe MGD include (1) increased tear volume, and (2) increased tangential redistribution of the tear film. It has been suggested that compensatory reflex tearing could lead to increased tear volume, accounting for the increased TMH in MGD.^30^^,^^31^ However, tear volume was not measured in the study by García-Marqués et al.,^30^ and in the study by Robin et al.,^31^ Schirmer test scores (without anesthesia) were normal in both the MGD and non-MGD cohorts, and not higher in the MGD cohort. Similarly, in our study, there was no evidence for increased tear production in the severe MGD cohort—mean Schirmer without anesthesia scores were the same (16.4 mm) in both the non-MGD and severe MGD cohorts.^32^ Therefore, the increased TMH in the severe MGD cohort probably reflects increased tangential redistribution of the tear film.

**Figure 3.**
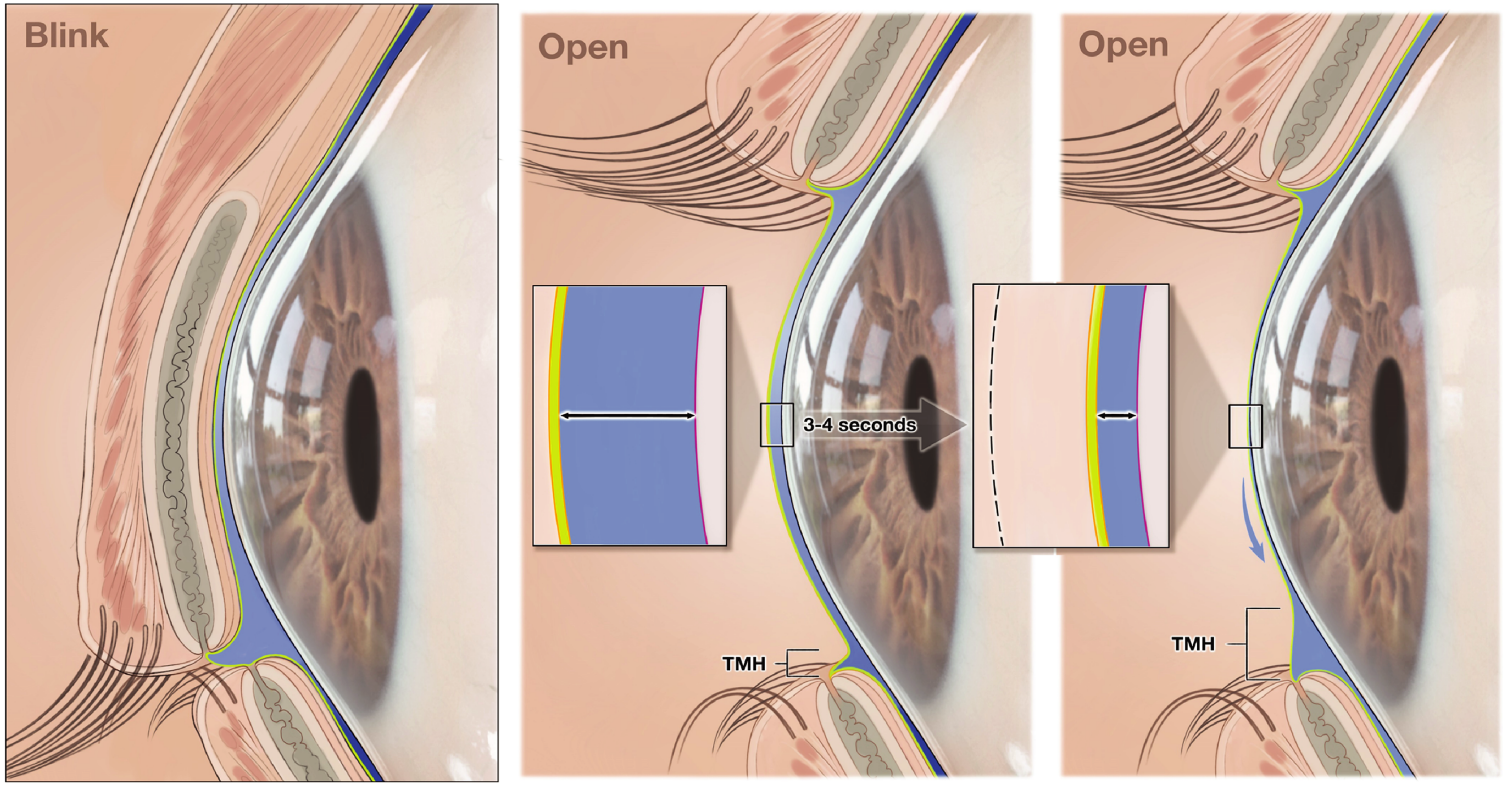
Illustration of changes in tear film dynamics after blinking. *Insets* show the lipid layer (*yellow*) and muco-aqueous layer (*blue*) of the tear film, with thinning of the muco-aqueous layer as the TMH increases during the inter-blink interval. TMH, tear meniscus height.

**Figure 4.**
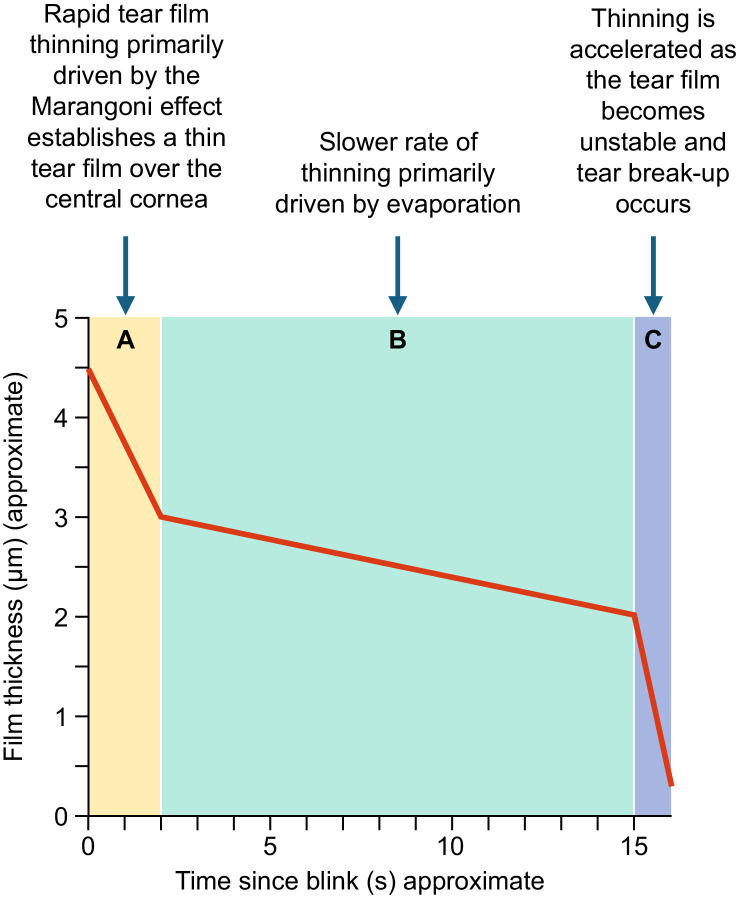
Three proposed phases of tear film thinning in the inter-blink interval. It is proposed that thinning in phase A is driven largely by the Marangoni effect and tangential flow toward the menisci, whereas thinning in phase B is driven primarily by evaporation. The rapid rate of thinning in phase C reflects tear film instability and break-up.

As the rate of central muco-aqueous layer thinning that we observed in healthy eyes is too rapid to be due to evaporation alone, we propose that muco-aqueous layer thinning measured by the TFI in the few seconds after a blink is primarily caused by tangential redistribution of the muco-aqueous layer from the central cornea to the menisci, which results in an increase in TMH during the inter-blink period (Fig. 3). We propose that tear film thinning during the inter-blink interval is triphasic (Fig. 4). In the first phase (phase A), there is rapid thinning from tangential flow preferentially toward the inferior meniscus, driven primarily by the Marangoni effect, with gravity and other forces, such as capillary suction pressure, playing a minor role.

In vivo, when the eye opens during a blink, the high lipid concentration at the lower lid margin causes reduced surface tension that results in tear film spreading, with the lipid layer and the muco-aqueous layer flowing upward by the Marangoni effect.^33^^,^^34^ The tear film over the central cornea continues to thin rapidly through phase A, until gradients in surface tension causing muco-aqueous layer spreading toward the menisci have dissipated. At the end of phase A, which may last approximately 2 to 3 seconds, a thin tear film has been established over the central cornea.

The second phase of muco-aqueous layer thinning after a blink (phase B) is characterized by a slower rate of thinning, which is driven mainly by evaporation. In the final phase (phase C), thinning is accelerated as the tear film becomes unstable and tear break-up occurs. This model is consistent with the suggestion by Nichols et al.^23^ that slow tear film thinning may be due to evaporation, whereas other mechanisms such as Marangoni flow are involved in rapid tear film thinning. The model suggests that tangential flow during phase A sets the initial tear film thickness present at the start of phase B, and changes in the composition of the lipid layer affecting surface tension may alter tangential muco-aqueous layer flow in the early period after a blink, making the eye more or less susceptible to tear breakup.

The role of tangential flow in tear film thinning has been relatively understudied. The TFI is the first marketed interferometer that can measure dynamic changes in the muco-aqueous layer of the tear film that occur in the first few seconds after blinking (in phase A). The rate of tear film thinning during this period is critical because it establishes the thickness of the tear film at the initiation of phase B. Evaporation has a background role in tear film thinning throughout the inter-blink interval, but it is the dominant mechanism of thinning in phase B.

Tangential flow of the muco-aqueous layer toward the menisci leads to a downward shift in the muco-aqueous layer from gravitational force. The muco-aqueous layer is redistributed to the inferior meniscus, with constant flow out of the inferior meniscus through the nasolacrimal duct pathways. This tangential redistribution of the muco-aqueous layer is significantly increased in eyes with MGD, with an increasing rate of muco-aqueous layer collapse (evident by the increase in TMH) associated with worsening MGD severity. We propose that in MGD, the TFLL is less able to prevent the collapse of the tear film, and this can be observed as an increased rate of muco-aqueous layer thinning (which has not yet been demonstrated) and an increase in TMH.

MGD-associated DED has traditionally been characterized as an evaporative DED, and the etiology clearly involves an increase in evaporation of the tear film. The rate of evaporation from the ocular surface has been shown to be increased in evaporative DED, with evaporation leading to tear hyperosmolarity and subsequent tissue damage.^35^ In the review of tear film dynamics by Tomlinson et al.,^36^ the mean tear evaporation rate was 13.57 nm/s in studies of healthy subjects and 21.05 nm/s in studies of subjects with DED (17.91 nm/s in subjects with aqueous-deficient DED and 25.34 nm/s in subjects with evaporative DED). However, characterization of MGD-associated DED as evaporative DED may be an oversimplification, with other mechanisms playing major roles in the tear film thinning that leads to tear film instability and break-up in MGD. Although changes in the composition and structure of the TFLL are believed to contribute to tear film instability and break-up in MGD-associated DED^6^^,^^7^ and the main function of the TFLL was traditionally believed to prevent evaporative loss, a more contemporary perspective is that its main purpose is to facilitate tear film spreading and prevent its collapse into the tear menisci.^37^

The main limitations of the present study are the small sample size (only 17 patients with measurable MALTR) and the comparison of the observed MALTR to evaporation rates reported in the literature rather than to evaporation rates measured in the same eyes under similar conditions. Further, the tear film interferometry measurements were done only in healthy controls, and there were no measurements of tear film dynamics such as MALT, MALTR, and LBUT in subjects with MGD. Other limitations include the absence of tear osmolarity data, the lack of tear film interferometry and TMH measurements in the same eyes, and the limited racial diversity of the study population.

A future study assessing MALTR, TMH (upper and lower), and ventilated-chamber evaporation in the same eyes under controlled relative humidity and temperature conditions is recommended to evaluate the relative contributions of tangential flow and evaporation to phase A and phase B tear film thinning. A study in subjects with and without MGD that uses both advanced tear film interferometry and measurements of TMH at defined timepoints after blinking is also warranted to confirm a faster MALTR associated with larger TMH in subjects with MGD. Further, TFI technology is evolving and could be used in future studies to evaluate tear film dynamics over a larger area of the ocular surface.

In summary, traditional theories of tear film dynamics propose evaporation as the primary driver of tear film thinning between blinks, but our study using the TFI highlights a major role for other mechanisms of tear film thinning. We believe the historical emphasis on evaporation is an oversimplification. Tear film thinning is not a function of evaporation alone but instead involves dynamic movement of the tear film layers, intricate interactions with the ocular surface, and blinking mechanics. One major mechanism that our work has highlighted is the importance of tangential flow of the tear film during the inter-blink period in tear film dynamics. The assertion that tangential flow and redistribution of the muco-aqueous layer (predominantly driven by the Marangoni effect with gravity and other forces playing a minor role) may be the dominant mechanisms for tear film thinning during the early period after blinking in healthy subjects requires additional follow-up studies. However, these initial observations have major implications for our understanding of the mechanisms of MGD-associated DED, and they may potentially help guide the development of novel therapeutic options for dry eye.
